# Compulsive exercise and mental health challenges in fitness instructors; presence and interactions

**DOI:** 10.1186/s40337-021-00446-0

**Published:** 2021-09-02

**Authors:** Christina Gjestvang, Solfrid Bratland-Sanda, Therese Fostervold Mathisen

**Affiliations:** 1 Department of Sports Medicine, Norwegian School of Sports Sciences, Oslo, Norway; 2 Department of Sports, Physical Education and Outdoor Studies, University of Southeast Norway, Kongsberg, Norway; 3 Faculty of Health, Welfare and Organisation, Østfold University College, P.O. Box 700, 1757 Halden, Fredrikstad, Norway

**Keywords:** Dysfunctional exercise, Compulsive exercise, Disordered eating, Depression, Anxiety, Physical activity, Fitness centers, Personal trainer, Group instructor, Eating disorders

## Abstract

**Background:**

Some physically active people exercise compulsively, which can be associated with several mental health challenges. Fitness instructors are considered important role models for an active, healthy lifestyle; yet little is known about their exercise motives and mental health. The aim of this study was to examine the presence of compulsive exercise and mental health challenges, and their interaction, in fitness instructors.

**Methods:**

A total of 270 fitness instructors from Norwegian fitness clubs were recruited for this cross-sectional study. Inclusion criteria were operating as instructors within the current year and understanding Norwegian language. Data were collected by an electronic questionnaire and included demographic information, hours of classes instructed and of personal physical activity, Compulsive Exercise Test (CET), Symptom Check List – 10 (SCL-10), Beck Depression Inventory (BDI), and Eating Disorder Examination Questionnaire (EDE-Q)).

**Results:**

Females had higher CET scores than males, and 9% of all respondents had CET score above clinical cutoff. Respondents with clinical CET score had higher SCL-10, BDI and EDE-Q global- and subscale scores compared with their counterparts. Although CET was positively and significantly associated with BDI, SCL-10, and EDE-Q, only the latter explained the CET score (ß = 1.23, 99% CI = 0.87, 1.59).

**Conclusion:**

About one out of eleven instructors were above clinical CET cut-off, revealing symptoms of compulsive exercise. EDE-Q significantly contributed in a model explaining 43% of the variation of compulsive exercise.

## Plain English summary

For some people, physical activity may be driven by compulsive thoughts, which can be associated with mental health issues and psychosocial impairment. This makes it important to understand the underpinnings to compulsive exercise (CE). Fitness instructors are considered important facilitators for a healthy and physically active lifestyle, but there is little knowledge on the occurrence of CE among these role models. Previous findings report on a significant frequency of symptoms of eating disorders in fitness instructors, and as such it is reasonable to assume that CE is present. By concurrently obtaining information on symptoms of depression, anxiety and eating disorders, this study was able to explore explanations to the variance in occurrence of CE. Our main findings were a moderately elevated frequency of symptoms of depression and anxiety, and that 9% of 270 male and female fitness instructors demonstrated CE behavior. Participants demonstrating CE had higher levels of symptoms of depression, anxiety, and eating disorders compared with those without CE. Nevertheless, only symptoms of eating disorders explained the variation in CE severity. Other than increasing our understanding of CE, the main implication from these findings is a need to increase mental health literacy in fitness instructors.

## Background

Fitness instructors (group training instructors and personal trainers) are exercise professionals considered as important facilitators for adaptation and maintenance of a healthy and physically active lifestyle in fitness club members [1–3]. Contrasting to this important role as healthy ideals and motivators, previous studies have found prevalence of 17 to 59% of eating disorders or disordered eating behavior among fitness instructors [4, 5]. Such unhealthy behavior has been associated with high exercise load [6] and compulsive exercise [4] and thus call for concern.

It has been debated whether compulsive exercise can occur without the presence of disordered eating or eating disorders. Prevalence of compulsive exercise, which is defined as driven and rigid exercise routines performed to avoid the discomfort of exercise deprivation, and which negatively interfere with daily life and social functioning [7], vary from 0.5–42% depending upon population examined. One previous finding suggested that excessive exercise, as defined in Diagnostic and Statistical Manual of Mental Disorders, does not associate to clinical psychosocial impairment without the presence of eating disorder psychopathology [8]. Another study found compulsive exercise to associate with depression and anxiety in professional and recreational exercisers, unfortunately this study did not assess eating disorder psychopathology and explanatory means to compulsive exercise [9]. As such, to our knowledge, there is lack of studies that have measured the construct *compulsive exercise* in exercising populations, such as fitness instructors, while concurrently obtaining information on symptoms of depression, anxiety and of eating disorders. Also, there is a knowledge gap regarding the contribution of depression, anxiety, and eating disorders in explaining the compulsive exercise.

In people with eating disorders, predictors of compulsive exercise include weight and shape concerns, affect regulation, compulsiveness, perfectionism and rigidity [10]. Further, athletic-ideal and thin-ideal internalization have been found as predictors of compulsive exercise in a female student population [11]. Depression and anxiety are common comorbid disorders among people with eating disorders and have also been associated with compulsive exercise [7], and thus it is of interest to explore how depression, anxiety, and eating disorders contribute in explaining compulsive exercise.

The objective of this study was to examine compulsive exercise and its relation to symptoms of depression, anxiety and of eating disorders in fitness instructors. Our research questions were as follows: 1) what is the prevalence of compulsive exercise, anxiety, and depression in fitness instructors, 2) is compulsive exercise correlated with symptoms of depression, anxiety, and of eating disorders), and 3) do the symptoms of anxiety, depression or eating disorders explain the variation in compulsive exercise in fitness instructors?

## Methods

The current results are a secondary analysis from a cross-sectional study in which fitness instructors in Norway were recruited between November 2019 and March 2020 [5]. The study aimed to measure body figure idealization and experiences of body appearance pressure, routines and motives for diet and physical activity, and mental health challenges. Participants responded to an electronic questionnaire estimated to take 40 min to respond to, with opportunities to pause.

### Participants

Participants recruited for this survey were operating fitness instructors (either personal trainers and/or group exercise instructors) in Norwegian fitness clubs during 2019/2020. In an attempt to keep a count on response rate, we first distributed recruitment directly per email to chief executive officers (CEOs) in large fitness club chains listed by the Norwegian enterprise federation “Virke Trening” or those listed in other available official listings. The CEOs were asked to respond with the number of employees who then received the recruitment information and link for participation. Unfortunately, most CEOs did not provide the actual number of employees, we lost the opportunity for calculation of eligibility for the study and decided to also recruit more widely in social media. Inclusion criteria were to operate as an instructor within the current year and to speak and understand Norwegian language. In total 304 instructors responded to recruitment, and of these 270 (211 women and 59 men) were included in this study (the remaining did not respond to the questions relating to the aims and outcomes of this study).

### Questionnaires

Initially, all participants responded on questions pertaining demographic information; age, profession, educational level, body mass index (BMI), hours with instructing classes, and hours with personal physical activity.

#### Compulsive exercise test (CET)

The CET (current Cronbach’s α = 0.76) is a 24-item questionnaire with 5 subscales, intending to measure compulsive exercise cognitions, feelings and behaviour [12]. All items are measured on a Likert scale ranging from 0 (never true) to 5 (always true), scores on two items were reversed for the analysis so higher score indicated higher level of compulsive exercise for all items. Scores on subscales are presented as means, with total score calculated as sum of mean subscale score. A total mean score ≥ 15 suggested as a cut-off for symptoms of compulsive exercise. The questionnaire has been validated in a healthy and clinical sample (adults with eating disorders) and within a national clinical sample [13, 14].

#### Hopkins symptom check list version with 10 items (SCL-10)

The SCL-10 (current Cronbach’s α = 0.88) is the short version of the SCL-25 (originally short form of SCL-90), which validly and reliably measures symptoms of depression (six items) and anxiety (four items) [15]. The SCL-10 consists of 10 items rated on a Likert scale ranging from 0 to 4 (“*not bothered”, to “bothered a lot”)* and scored as a total mean score. A clinically meaningful cut-off of ≥1.85 has been previously suggested [15].

#### Beck depression inventory version 1a (BDI)

The BDI (current Cronbach’s α = 0.85) is a 21-item questionnaire measuring current (last 2 weeks) symptoms of depression, with items using a Likert scale ranging from 0 (not at all) to 3 (extreme) [16]. Total score ranges from 0 to 63, with a total score ≥ 21 indicating a clinically significant episode of major depression [16].

#### Eating disorder examination questionnaire (EDE-Q)

The EDE-Q (current Cronbach’s α = 0.94) is a 24-item questionnaire measuring symptoms of eating disorders [17]. It consists of four subscales and a global score (18 items rated with a Likert scale ranging from 0 (never) to 6 (every day) and includes 6 questions on frequency of eating disorder behaviour (binge eating and purging behaviour). Each scales is presented as a mean score, with a global score ≥ 2.5 indicating symptoms of an eating disorder, based on sensitivity and specificity from a clinical and a healthy sample in Norway [18].

### Statistics

Data were analyzed using SPSS (IBM Corp. Released 2016. IBM SPSS Statistics for Windows, Version 24.0. Armonk, NY: IBM Corp). An independent t-test for continuous variables or chi-squared test for proportions were used as appropriate. Kruskal Wallis test was used for skewed data (EDE-Q). A multiple regression analysis was performed to explain CET total score. In the analysis, significant variables from Pearson’s correlation (SCL-10 total score and BDI total score) or Spearman’s rho (EDE-Q total score) analyses were entered. The model was adjusted for sex. Further, based on the results from the multiple regression analysis, in order to consider to what extent the observed association between compulsive exercise and the independent variables were mediated by sex, a moderator analysis was performed. Finally, we estimated the odds of having clinical symptoms for depression (BDI total score ≥ 21) or eating disorders (EDE-Q global score ≥ 2.5) according to having CET-scores below or above clinical cut-off. We also calculated Hedges *g* effect size, with values around 0.2, 0.5 and 0.8 interpreted as weak, medium and strong effect sizes, respectively. Results are presented as means ± SD (median ± interquartile range for EDE-Q), or frequencies (n) and percentages (%), correlations coefficient (r), beta coefficient (ß), standard errors, t-value, 99% CI for ß, odds ratio (OR), and effect sizes (Hedges g). A two-tailed alpha level of 0.01 was used for statistical significance.

## Results

A total of 270 (men, *n* = 59) fitness instructors answered the questionnaire. Of these, 181 (67%) worked as group exercise instructors, 36 (13%) as personal trainers, while 53 (20%) reported both professions. Descriptive data for the included fitness instructors is presented in Table 1.

**Table 1:** Descriptive data among Female and Male Fitness Instructors

Variable	All (*n* = 270)	Men (*n* = 59)	Women (*n* = 211)	Mean diff.	95% CI mean diff.	*p*
	Mean ± SD	Mean ± SD	Mean ± SD			
Age (years)	36.10 ± 11.92	39.19 ± 13.66	35.29 ± 11.28	3.90	0.46, 7.33	0.026
Body weight (kg)	69.00 ± 12.89	84.84 ± 8.51	64.57 ± 10.15	20.27	17.42, 23.12	<0.001
BMI (kg/m^2^)	23.58 ± 3.33	25.89 ± 2.43	22.93 ± 3.27	2.95	2.05, 3.85	<0.001
Personal exercise hours (not including instructing classes), hours/week	6.88 ± 7.26	6.59 ± 4.36	6.97 ± 7.90	0.37	1.17, 1.93	0.632
Instructing classes, hours/week	8.15 ± 10.58	9.45 ± 13.52	7.81 ± 9.62	1.64	1.43, 4.71	0.294
	n (%)	n (%)	n (%)			
High educational level (≥3 years of higher education)	172 (63.7)	33 (55.9)	138 (65.7)			0.290
Higher exercise education level (≥3 year of higher education)	64 (23.7)	14 (23.7)	50 (23.8)			0.856
Distribution of professions						0.946
Personal trainer	36 (13.3)	7 (11.9)	29 (13.8)			
Group exercise instructor	181 (67.1)	41 (69.5)	139 (66.2)			
Both professions	53 (19.6)	11 (18.6)	42 (20.0)			

*BMI* Body mass index

### Mental health challenges

Results from CET, SCL-10, BDI, and EDE-Q are presented in Table 2. Participants above CET cut-off were more likely to be above cut-offs for SCL (OR = 13.9, 95% CI 5.1 to 37.3), BDI (OR = 17.0, 95% CI 5.5 to 53.0), and EDE-Q (OR = 28.0, 95% CI 9.8 to 79.8). There were no sex differences in participants scoring above cut-off for CET, SCL-10, BDI, and EDE-Q.

**Table 2:** Numbers above clinical cut-offs and mean scores in Compulsive Exercise Test (CET), Symptom Check List (SCL-10), Beck Depression Inventory (BDI), and Eating Disorder Examination Questionnaire (EDE-Q) Scores among Female and Male Fitness Instructors

Variable	All (*n* = 270)	Men (*n* = 59)	Women (*n* = 211)	Mean diff.	95% CI mean diff.	*p*	Hedges *g*
	n (%)	n (%)	n (%)				
CET ≥ 15	24 (9)	2 (3)	22 (11)	-	-	0.228	-
SCL-10 ≥ 1.85	42 (16)	6 (10)	36 (17)	-	-	0.138	-
BDI ≥ 21	15 (6)	3 (5)	12 (6)	-	-	0.954	-
EDE-Q ≥ 2.5	29 (11)	2 (3)	27 (13)	-	-	0.030	-
	Mean ± SD	Mean ± SD	Mean ± SD				
CET. Total score	11.50 ± 2.39	10.58 ± 1.99	11.78 ± 2.41	1.20	0.53, 1.88	0.001	-0.52
CET, Subscales:							
*Avoidance*	1.65 ± 0.97	1.40 ± 0.79	1.72 ± 1.00	0.32	0.07, 0.57	0.010	-0.33
*Weight Control*	1.86 ± 0.91	1.64 ± 0.69	1.93 ± 0.95	0.29	0.07, 0.52	0.009	-0.32
*Mood Improvement*	4.03 ± 0.80	3.59 ± 0.93	4.16 ± 0.71	0.57	0.34, 0.79	0.000	-0.75
*Lack of Exercise Enjoyment*	0.77 ± 0.60	0.71 ± 0.64	0.79 ± 0.59	0.07	-0.10, 0.25	0.406	-0.13
*Exercise Rigidity*	3.17 ± 0.80	3.22 ± 0.86	3.16 ± 0.89	0.06	-0.31, 0.19	0.641	0.07
SCL-10, Total Score	1.47 ± 0.51	1.32 ± 0.45	1.52 ± 0.52	0.19	0.04, 0.34	0.012	-0.40
BDI, Total Score	6.34 ± 6.33	5.03 ± 5.65	6.71 ± 6.49	1.67	-0.15, 3.50	0.073	-0.27
EDE-Q, Global score	0.65 ± 1.13	0.44 ± 0.79	0.73 ± 1.21	0.29	-	0.020	-
EDE-Q, Subscales:							
*Weight concern*	1.17 ± 1.22	0.77 ± 0.84	1.29 ± 1.29	0.52	0.23, 0.81	0.000	-0.43
*Restraint*	0.98 ± 1.15	0.85 ± 1.03	1.02 ± 1.19	0.17	-0.16, 0.51	0.321	-0.15
*Eating concern*	0.43 ± 0.81	0.13 ± 0.32	0.52 ± 0.89	0.39	0.24, 0.55	0.000	-0.49
*Shape concern*	1.37 ± 1.31	0.94 ± 1.00	1.49 ± 1.37	0.55	0.22, 0.88	0.001	-0.42

EDE-Q total score is presented as median ± interquartile range

### Mental health challenges according to CET cut-off

Comparisons of mental health challenges according to CET cut-off are shown in Table 3. All mental health challenges were rated higher among participants above CET cut-off compared with participants below (*p* = < 0.01), with medium to large effects.

**Table 3:** Comparison of Mental Health Challenges according to CET Cut-off

Variable	Above cut-off (≥15) for CET total score. (*n* = 24)	Below cut-off (≤14) for CET total score. (*n* = 246)	Mean diff.	95% CI mean diff.	*p*	Hedges *g*
	Mean ± SD	Mean ± SD				
CET, Total score	16.56 ± 1.47	11.00 ± 1.83	5.55	4.79, 6.31	<0.001	3.09
CET, Subscales:						
*Avoidance*	3.63 ± 0.83	1.46 ± 0.74	2.16	1.85, 2.48	<0.001	2.90
*Weight Control*	3.45 ± 0.83	1.71 ± 0.76	1.73	1.41, 2.06	<0.001	2.27
*Mood Improvement*	4.48 ± 0.52	3.98 ± 0.81	0.49	0.25, 0.73	<0.001	0.63
*Lack of Exercise Enjoyment*	1.05 ± 0.69	0.74 ± 0.59	0.31	0.05, 0.56	0.017	0.52
*Exercise Rigidity*	3.94 ± 0.76	3.09 ± 0.86	0.84	0.48, 1.20	<0.001	1.00
SCL-10, Total Score	2.16 ± 0.66	1.41 ± 0.44	0.74	0.53, 0.96	<0.001	1.62
BDI, Total Score	16.13 ± 9.05	5.39 ± 5.10	10.73	-13.07, -8.40	<0.001	1.94
EDE-Q, Global score	2.67 ± 2.69	0.60 ± 0.99	2.07	-	<0.001	-
EDE-Q, Subscales:						
*Weight concern*	3.18 ± 1.42	0.98 ± 1.02	2.19	-2.67, -1.71	<0.001	2.07
*Restraint*	2.45 ± 1.70	0.84 ± 0.99	1.60	-2.07, -1.13	<0.001	1.51
*Eating concern*	1.86 ± 1.55	0.30 ± 0.55	1.55	-1.87, -1.24	<0.001	2.25
*Shape concern*	3.31 ± 1.48	1.19 ± 1.14	2.12	-2.65, -1.59	<0.001	1.81

EDE-Q global score is presented as median ± interquartile range

### Pearson’s correlation coefficients, spearman’s rho, and multiple linear regression

Correlations between CET total score and symptoms of depression, anxiety and of eating disorders are presented in Table 4, with the results from the second multiple linear regression presented in Table 5. For all participants, only EDE-Q global score was statistically significant in the multiple linear regression, explaining 43% of the variation in CET-total score, with a beta value of 1.23 (*p* = < 0.001). Finally, the moderator analysis revealed that sex did not moderate the relationship between CET total score and EDE-Q global score (R^2^ = 0.001, F = 0.534, *p* = 0.465).

**Table 4:** Pearsons’ Correlation (SCL-10 and BDI) and Spearman’s rho (EDE-Q) between CET Total Score and Mental Health Challenges

	CET total score	
Variable	All (*n* = 270)	Below CET cut-off (*n* = 246)
	*r*	*r*
**SCL-10, Total Score**	0.475^a^	0.325^a^
**BDI, Total Score**	0.467^a^	0.232^a^
**EDE-Q, Global score**	0.609^a^	0.542^a^

CET cut-off is defined ≥global score 15

^a^Correlations significant at the 0.01 level

**Table 5:** Multiple Regression Analysis Summary for Mental Health predicting CET Total Score

	ß^a^	SE	t	p	99% CI for ß	
Variable					Lower	Upper
**All (*n*= 270)**						
**Sex**	-0.43	0.28	-1.56	0.121	-1.15	0.29
**SCL-10, Total Score**	0.81	0.44	1.81	0.071	-0.35	1.96
**BDI, Total Score**	-0.002	0.036	-0.06	0.947	-0.10	0.09
**EDE-Q, Global score**	1.23	0.14	8.88	<0.001	0.87	1.59
**Constant**	9.64	0.63	15.27	<0.001	8.00	11.28
**Below CET cut-off (*n*= 246)**						
**Sex**	-0.37	0.25	-1.45	0.148	-1.03	0.29
**SCL-10, Total Score**	0.89	0.46	1.96	0.052	-0.29	2.08
**BDI, Total Score**	-0.05	0.04	-1.38	0.170	-0.15	0.05
**EDE-Q, Global score**	0.98	0.15	6.37	<0.001	0.58	1.39
**Constant**	9.71	0.62	15.55	<0.001	8.09	11.33

^a^Unstandardized ß

## Discussion

We aimed to study the presence of, and the relation between, compulsive exercise and symptoms of depression, anxiety and eating disorders, and to study explanation to the variation of compulsive exercise in fitness instructors. Our main findings were a moderately elevated frequency of symptoms of depression and anxiety (1 of 6 had symptoms of depression and anxiety), and that compulsive exercise was explained by symptoms of eating disorders but not by symptoms of depression or anxiety. We also found that respondents with a CET-score above clinical cut-off had higher intensity in symptoms of depression, anxiety, and eating disorders compared with respondents below cut-off.

Prevalence of compulsive exercise was comparable to previous studies from exercising populations [7], yet much higher compared with predictions from the general adult population (9% versus 0.5%) [19]. One could speculate whether the occupation of fitness instructors attracts people susceptible to compulsive exercise and eating disorders, as the prevalence of eating disorders also have been found higher compared to the general population [4, 5]. Another explanation may be that the fitness club environment possibly further facilitates compulsive exercise behavior. This may raise a concern since fitness instructors who function as role models should be healthy themselves, as they need to be able to communicate healthy attitudes to the fitness club members.

The findings of symptoms of depression and anxiety are in line with results from the general adult population in Norway (i.e. 16 to 22%) [20] and somewhat higher than former research among elite athletes, yet error estimates require caution with conclusion of differences between instructors and athletes [21, 22]. The associations between compulsive exercise and symptoms of depression and anxiety confirm findings from previous studies [9, 10, 13, 23, 24]. Still, we identified an odds ratio of 14–28 times higher risk of clinical levels of anxiety, depression and eating disorders among instructors with compulsive exercise. This adds strength to the knowledge of compulsive exercise as a dysfunctional behavior occurring with other mental health challenges.

Although compulsive exercise correlated with anxiety, depression, and eating disorders in the current study, it was only explained by presence of eating disorder symptomatology. Here, sex did not moderate this finding. Symptoms of anxiety and depression are also common comorbidities with eating disorders [25], and it is therefore difficult to extract these features in examination of compulsive exercise. Thus, we checked the analysis for potential multicollinearity and found that this was not an explanation for this finding. It is important though, to consider that the results might be influenced by methodological limitations with the instruments (i.e., lack of validation in this population), or different sensitivity of the instruments included in the current study. For example, the EDE-Q could be a more sensitive instrument for eating disorders, previously demonstrated by its validation [26], than the SCL-10 is for depression and anxiety (it measures two constructs within one questionnaire with rather few questions). This is underlined by the relatively high frequency of mental health symptoms evaluated by the SCL-10 (i.e. 16%), and the lower frequency of symptoms of depression evaluated by the BDI (i.e. 6%).

The current findings suggest a higher frequency of participants with a clinical score of SCL-10 compared to previous studies in adult samples from the general population [15]. This contrasts to the current lower frequency with clinical BDI-score compared with normative values from samples of comparable age [27]. These findings can possibly be understood by the strong evidence-base of the effects from physical activity in both prevention and treatment of depression (measured specifically by BDI), still weaker evidence for this effect in anxiety (measured by SCL-10) [28].

Nevertheless, the occurrence of compulsive exercise, anxiety and eating disorders identified in this study calls for concern for the health and wellbeing of fitness instructors. Additionally, as the fitness instructors are expected to be healthy role models, it would be beneficial for future studies to examine how compulsive exercise and mental health challenges within fitness instructors might affect their communication and impact on clients. Implications from these findings, are a need to increase mental health literacy, and specifically regarding healthy exercise motives and symptoms of eating disorders. To date, these issues have not been specifically addressed in detail in the curriculum of fitness instructors, at least not in Norway.

### Strengths and limitations

To our knowledge, no studies have investigated mental health challenges such as anxiety and depression in a fitness instructor population. A relatively large sample size of 270 participants, the inclusion of both sexes, and an electronic questionnaire primarily based on previously validated instruments may be considered strong aspects of our study. Study limitations are an assumingly low response rate and that we were unable to calculate initial eligibility. Thus, we do not know to what extent fitness instructors who agreed to participate are representative of Norwegian instructors. Also, another limitation is the cross-sectional design and that we could not establish the direction of the observed associations. Finally, the sample size with participants scoring *above* CET cut-off (*n* = 24) was not large enough for statistical analyses. Our quantitative design with numeric results may also be too narrow to explain the complex aspect of compulsive exercise and mental health.

## Conclusion

In conclusion, one out of eleven- fitness instructors were above CET cut-off, revealing symptoms of compulsive exercise. Compulsive exercise was associated with symptoms of anxiety, depression and eating disorders, whereas only symptoms of eating disorders was significant in the model explaining the variation in compulsive exercise. Findings suggest a need for increased mental health literacy among fitness instructors.

## Data Availability

The datasets used and/or analyzed during the current study are available from the corresponding author on reasonable request.

## Abbreviations

BDI: beck depression inventory
BMI: body mass index
EDE-Q: eating disorder examination questionnaire
CE: compulsive exercise
CEO: chief executive officers
CET: compulsive exercise test
SCL-10: symptom check list, ten items
